# DNA Compass: a secure, client-side site for navigating personal genetic information

**DOI:** 10.1093/bioinformatics/btx135

**Published:** 2017-03-11

**Authors:** Charles Curnin, Assaf Gordon, Yaniv Erlich

**Affiliations:** 1 New York Genome Center, New York, NY, USA; 2 Department of Computer Science, Fu Foundation School of Engineering, Columbia University, New York, NY, USA; 3 Center for Computational Biology and Bioinformatics, Columbia University, New York, NY, USA

## Abstract

**Motivation:**

Millions of individuals have access to raw genomic data using direct-to-consumer companies. The advent of large-scale sequencing projects, such as the Precision Medicine Initiative, will further increase the number of individuals with access to their own genomic information. However, querying genomic data requires a computer terminal and computational skill to analyze the data—an impediment for the general public.

**Results:**

DNA Compass is a website designed to empower the public by enabling simple navigation of personal genomic data. Users can query the status of their genomic variants for over 1658 markers or tens of millions of documented single nucleotide polymorphisms (SNPs). DNA Compass presents the relevant genotypes of the user side-by-side with explanatory scientific resources. The genotype data never leaves the user’s computer, a feature that provides improved security and performance. More than 12 000 unique users, mainly from the general genetic genealogy community, have already used DNA Compass, demonstrating its utility.

**Availability and Implementation:**

DNA Compass is freely available on https://compass.dna.land.

## 1 Introduction

We have entered the era of ubiquitous genomic information. Today, approximately three million people worldwide have access to their genome-wide autosomal information via direct to consumer (DTC) genomic companies such as 23andMe, AncestryDNA, and FamilyTreeDNA (Khan and Mittelman, 2013). Recent studies have predicted that by 2025, at least 100 million individuals will have their genomes sequenced (Stephens *et al.*, 2015) and whole genome sequencing will become a routine part of newborn screening (Burn and Flinter, 2013).

Interpretation of this data, however, remains difficult. While recent ethics studies have highlighted the importance of returning results to research participants, most studies are reluctant to provide any interpretation due to regulatory complications (Jarvik *et al.*, 2014; Evans and Rothschild, 2012). As an alternative, a growing number of entities, including DTC companies or Genes for Good, return raw data to participants. However, the scale of genomic information precludes even simple analysis by people without knowledge of bioinformatics. VCF files of personalized genomic tests can reach gigabytes of data; although these files are textual and human-readable, they are largely inaccessible to individuals who lack basic command-line skills. We encountered this problem recently in connection with our website DNA.Land (https://dna.land) (Erlich, 2015), which crowdsources genomic datasets directly from people who were tested by DTC companies.

One of the features of DNA.Land is genomic imputation of the user’s DTC file to report 39 million variants. We anticipated that this unique feature would be well-received by the 30 000 participants of DNA.Land. However, most participants who downloaded the data expressed a high level of frustration after repeatedly crashing their Excel spreadsheet or word processor when attempting to open the imputed VCF file.

Here, we present DNA Compass (https://compass.dna.land), a free website that enables the navigation of personal genomic information without command-line knowledge. Our website aims to empower the growing number of individuals who have access to their genomic datasets but are uninterested in pursuing a bioinformatics or computer science degree just to search for a particular SNP or trait in their raw data. To test the website, we announced its launch on the Facebook pages of several genetic genealogy groups. We had nearly 2500 unique users within the first 2 days of operation, demonstrating the wide interest in such a tool. Since launching, we have had more than 13 000 unique users. Overall, we received very positive feedback.

## 2 User experience

To use DNA Compass, a user must specify two files: a compressed VCF file and a corresponding Tabix index file (Danecek *et al.*, 2011). We selected these formats due to their wide popularity: they are reported to DTC participants on DNA.Land and are popular in whole genome sequencing projects. DNA Compass supports GRCh37 and GRCh38 (with newer genome builds once available).

DNA Compass quickly processes the genomic information and validates the format. First, the site infers the genome build version from the decompressed VCF file’s header. If the file’s build version cannot be determined, the site alerts the user and attempts to extract the genotypes according to GRCh37hg19. If a user selects a VCF with an unsupported genome build, an error message is presented.

Once the data is retrieved from the VCF file, the user can specify an individual SNP by rsID or enter a particular condition/trait (e.g. height or lupus) from more than 1658 categories. To assist the user to find the topic of interest quickly, the website completes the text as the user types and also offers the full list of categories. Next, DNA Compass presents a table with the desired information. For each SNP, we present the following information: the user’s genotype, chromosome, cytoband, risk allele and effect size. Risk allele and effect size information is based on the GWAS Catalog of the European Bioinformatics Institute (EBI).

**Fig. 1.**
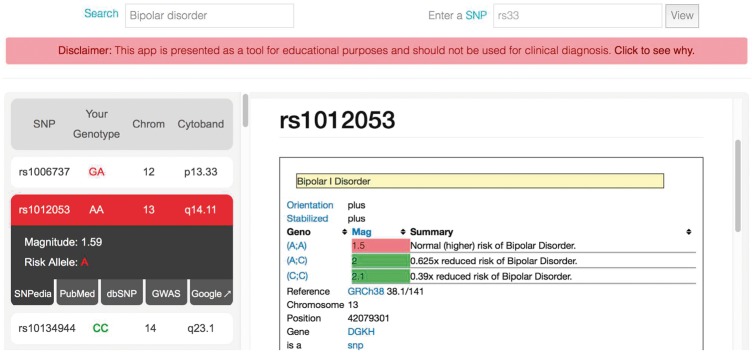
DNA Compass report for some of the height-associated genotypes of a user (left) along with information from SNPedia (right). By clicking on each SNP in the list (left), the user can select relevant information related to the SNP from SNPedia, PubMed, dbSNP, GWAS Central or Google. The search field (top left) supports over 1658 markers. The SNP field (top right) allows the query of tens of millions of variants. The data presented belongs to the senior author of this manuscript

Importantly, the website does not directly interpret genomic information. Instead, for each SNP, users can navigate five publicly available resources, namely SNPedia (Cariaso and Lennon, 2012), PubMed, dbSNP (Sherry *et al.*, 2001), GWAS Central (Beck *et al.*, 2014) and Google. With the exception of Google, clicking on any of these resources opens an iframe window that presents the selected website side-by-side with the user’s own genetic information (Fig. 1). Crucially, we do not send any private information to any of these websites. This model allows the user to access relevant scientific knowledge with maximum efficiency. If the user chooses the Google search option, a new tab is opened in the browser where the search term is the SNP’s rsID.

In addition to presenting the data in tabular format, the user can also navigate the SNPs through a digital karyotype diagram.

There are several options for user support. First, an example VCF-Tabix pair is available for those who want to test the website. In addition, we include an extensive FAQ section and guide. Finally, the website is dynamic and provides quick feedback for the user’s actions.

## 3 Architecture and compatibility

Aside from their popularity, we chose the VCF and Tabix file format because a compressed VCF can be stored and managed easily, while the Tabix index file makes it easy to navigate to and decompress slices of the VCF. DNA Compass can accept nearly any valid VCF file, including those that are not from DNA.Land. The site can even process VCF files which contain nonstandard or inaccurate rsIDs or SNP markers—even files which contain no markers at all—as long as the chromosome and position information is accurate.

DNA Compass operates almost exclusively on the client-side. The sole role of the server is to provide static webpages and information regarding SNP positions and associations. Importantly, the client never transmits information from the user’s VCF and Tabix files. This has two advantages. First, it obviates the need for transferring massive VCF files across the web, which means a quick response to user queries. Tests with a standard Macbook Air laptop show that it takes a few seconds on either Chrome or Firefox to return the results of a query. Second, by restricting the processing of data to the client side, we mitigate genetic privacy issues related to the management and storage of personal genomic data (Erlich and Narayanan, 2014).

The server-side uses DreamFactory and SQLite3 to store the coordinates (chromosome and position in GRCh37/hg19 and GRCh38/hg38) for each SNP rsID in dbSNP141. The client-side functionality employs jQuery, Bootstrap, D3.js and jsf-local-aerial. To facilitate future developments, we also released the entire source code on GitHub (https://github.com/TeamErlich/dna-land-compass) under the BSD license.

## 4 Conclusion

With the advent of large-scale public efforts to making DNA sequencing more accessible, a massive number of individuals with genomic data, and the increasing integration of genomics in medicine, we envision a growing demand from the general public for technological solutions that will allow them navigate their raw data. DNA Compass aims to increase genetic literacy and empower research participants to understand their personal genomic data and advocate for themselves.
